# Differences between native and prosthetic knees in terms of cross-sectional morphology of the femoral trochlea: a study based on three-dimensional models and virtual total knee arthroplasty

**DOI:** 10.1186/s12891-017-1529-x

**Published:** 2017-04-20

**Authors:** Zhe Du, Shichang Chen, Mengning Yan, Bing Yue, You Wang

**Affiliations:** 1 Department of Bone and Joint Surgery, Renji Hospital, School of Medicine, Shanghai Jiaotong University, 145 Middle Shandong Road, Shanghai, 200001 China; 2 Department of Orthopaedic Surgery, Ninth People’s Hospital, Shanghai Jiaotong University School of Medicine, Shanghai, China

**Keywords:** Total knee arthroplasty, Femoral trochlea, Morphology, Prosthesis design

## Abstract

**Background:**

The cross-sectional morphology of the prosthetic knee is crucial to understanding patellar motion and quadriceps strength after total knee arthroplasty. However, few comparative evaluations of the cross-sectional morphology of the femoral trochlea have been performed in the native knee and currently available femoral implants, and the relationship between the trochlear anatomy of prosthetic components and post-operative patellofemoral complications remains unclear. We aimed to investigate the differences in cross-sectional morphology of the femoral trochlea between native knees and prosthetic femoral components.

**Methods:**

Virtual total knee arthroplasty was performed, whereby four different femoral components (medial-pivot, Triathlon, NRG and NexGen) were virtually superimposed onto three-dimensional models of 42 healthy femurs. The following morphological parameters were measured in three cross-sections (0, 45 and 90°) of the femoral trochlea: sulcus height, lateral tilt angle, medial tilt angle and sulcus angle. Only statistically significant differences are described further (*p* < 0.05).

**Results:**

In the 0° cross-section, sulcus height was smaller in the native knee than in the Triathlon, NRG and NexGen components; all prosthetic components had smaller lateral tilt angles and larger medial tilt angles. In the 45° cross-section, sulcus height was larger in the native knee than in the medial-pivot, Triathlon and NexGen components; both lateral and medial tilt angles were smaller in the prosthetic components. In the 90° cross-section, sulcus height was smaller in the native knee than in the medial-pivot component; all prosthetic components had a larger lateral tilt angle and smaller medial tilt angle. In all cross-sections, the sulcus angle was smaller in the native knee.

**Conclusions:**

The discrepancy between native and prosthetic trochlear geometries suggests altered knee mechanics after total knee arthroplasty, but further cadaveric, computational or fluoroscopic investigations are necessary to clarify the implications of this observation. Our findings can be used to optimize biomechanical guidelines for total knee arthroplasty (patellar resurfacing or non-resurfacing) in Chinese individuals so as to decrease the risk of patellar lateral dislocation, to maintain stability and to optimize extensor kinematics.

## Background

The most common complications after total knee arthroplasty (TKA) are related to femoropatellar problems, with residual pain in the anterior knee manifested in 5–45% of patients [1–3]. It has been proposed that excessive quadriceps load and altered patellar kinematics contribute to the development of patellar complications after TKA [3, 4]. Previous findings suggest that limitations of the implant design may result in such complications [5–7], and numerous authors have emphasized the changes in knee kinematics following TKA. Merican et al. [8] noted that TKA led to significant changes in patellofemoral kinematics, with significant increases in lateral shift, tilt and rotation compared to those characteristic to the native knee. Similarly, Akbari et al. [9] reported that the postoperative patella was more inferiorly positioned and tilted laterally in mid-flexion. It was speculated that these kinematic changes were due to trochlear dysplasia, since appropriate design for the prosthetic trochlea was accepted as the main determinant of patellofemoral outcome in TKA [10]. Previous evidence of trochlear dysplasia in the design of the femoral component was obtained based on 14 digital TKA models, but these findings were not evaluated in the context of the clinical outcomes achieved with the evaluated implants [7]. Saffarini et al. [11] highlighted the influence of patellofemoral geometry on mid-flexion kinematics after comparing two different knee components (HLS Noetos® and KneeTec®). In their simulation study, Varadarajan et al. [6] also measured the trochlear geometry before and after TKA, but did not discuss the effect of TKA on patellar motion and soft-tissue changes. To our knowledge, few comparative evaluations of the cross-sectional morphology of the femoral trochlea have been performed in the native knee and currently available femoral implants, and the relationship between the trochlear anatomy of prosthetic components and post-operative patellofemoral complications remains unclear.

In the present study based on virtual TKAs, whereby femoral implants were superimposed onto three-dimensional (3D) models of healthy femurs, we provide a detailed comparison between native and prosthetic knees regarding the cross-sectional morphology of the femoral trochlea, evaluated in terms of the sulcus height (H), lateral tilt angle (α), medial tilt angle (β) and sulcus angle. We aimed to investigate potential differences between the native knee and currently available prosthetic knee designs, and subsequently analyze the effects of prosthetic trochlear design on quadriceps strength and retinacula tension following TKA. The findings of our study are relevant for optimization of implant design, patient diagnosis and surgical technique. Our original hypothesis was that patellar kinematic changes and quadriceps weakness after TKA were due to irrational prosthetic trochlear design.

## Methods

The study included 42 healthy Chinese participants (10 male and 32 female), with an average age of 45.8 years (range, 34–57 years), an average height of 161.4 cm (range, 150–179 cm), an average body mass index of 23.7 kg/m^2^ (range, 16.5–29.6 kg/m^2^), and an average mechanical axis of the lower limb of 179.7° (range, 174.7–184.4°). Only participants with healthy knees were included. The exclusion criteria were previous knee trauma or knee pain; soft tissue injury; osteoarthritis; and other chronic diseases of the musculoskeletal system.

### Obtaining the 3D models of the native and prosthetic knee

Computed tomography (CT) images (Light speed 16; GE Medical Systems, Milwaukee, WI) were used to create 3D knee models. Only models of the right knee were included in the analysis. To reduce radiographic exposure, CT slices were acquired at intervals of 0.6 mm for the knee joint, and at intervals of 2 mm for the hip and ankle joints (resolution, 512 × 512 pixels). Four types of prosthetic components were evaluated in the present study, namely the Advance Medial-Pivot (MP) Knee System, (MicroPort Orthopedics Co., Arlington, TN), the Triathlon® Knee System (Stryker Co., Kalamazoo, MI), the NRG® Knee System (Stryker Co., Mahwah, NJ) and the NexGen® Complete Knee Solution (Zimmer Inc., Warsaw, IN). A 3D laser scanner (KLS-171; Kreon Technologies, Limoges, France) was used to create 3D models of the metal femoral components for the right knee. CT and laser scanning data were imported into Geomagic Studio version 10.0 (Geomagic Inc., Research Triangle Park, NC) in order to reconstruct the 3D models of the native knee and prosthetic components, respectively.

### Selecting a suitable size of the prosthetic component for virtual TKA

**Fig. 1:**
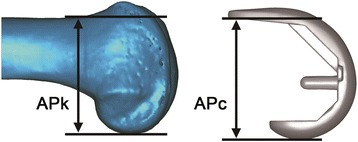
Approach for selecting prosthetic components for performing virtual total knee arthroplasty. The size of the femoral component used in the simulation was chosen so that the three-dimensional models of the native knee and prosthetic components have similar anteroposterior dimensions in the sagittal plane (|APk – Apc| < 2 mm; APk, distance between the anterior femoral cortex and posterior condyle in the model of the native femur; APc, distance between the most proximal point of the backside of the anterior flange and the posterior condyle in the model of the prosthetic component)

According to the general principles of TKA, the size of the femoral component was chosen based on the difference between the anteroposterior dimensions of the 3D models of the knee and prosthetic component in the sagittal plane [12]. In the model of the native knee, the anteroposterior dimension APk was defined as the distance between the anterior femoral cortex and the posterior condyle. In the model of the prosthetic component, the anteroposterior dimension APc was defined as the distance between the most proximal point of the backside of the anterior flange and the posterior condyle (Fig. 1). The criterion for selecting an appropriate size of the prosthetic component was set at |APk – Apc| < 2 mm. For example, if APk was 54.5–58.5 mm, an MP component of size 3 (APc = 56.5 mm) was selected, whereas if APk was < 54.5 mm or > 58.5 mm, MP components of size 2 or 4, respectively, were selected.

### Superimposing the prosthetic component model onto the knee model

We performed virtual surgery (virtual TKA) with the purpose of detecting the appropriate size of the implant (i.e., to size the implant). The following 3D planes were established for the virtual TKAs. The coronal plane was defined as the plane passing through the farthest posterior points of the medial and lateral condyles and those of the greater trochanter. The sagittal plane was defined as the plane passing through the center of the femoral head and the center of the intercondylar notch of the knee, perpendicularly to the coronal plane. The transverse plane was defined as the plane perpendicular to both the coronal and sagittal planes. The model of the selected prosthetic component was then positioned with its coronal plane parallel to the coronal plane of the native-knee model. Next, the model of the prosthetic component was oriented with its distal condyles parallel to the transverse plane, and its posterior condyles at 3° of external rotation from the coronal plane of the native-knee model. The model of the prosthetic component was translated in the 3D space until it overlapped with the native-knee model, such that: the medial-lateral center of the prosthetic component reached the sagittal plane of the native knee; the backside of the most proximal point of the anterior flange of the prosthetic component reached the anterior cortex of the native femur; and the distal medial condyle of the prosthetic component reached the surface of the medial condyle of the native knee [12].

### Cutting planes and parameters of the cross-sectional morphology

**Fig. 2:**
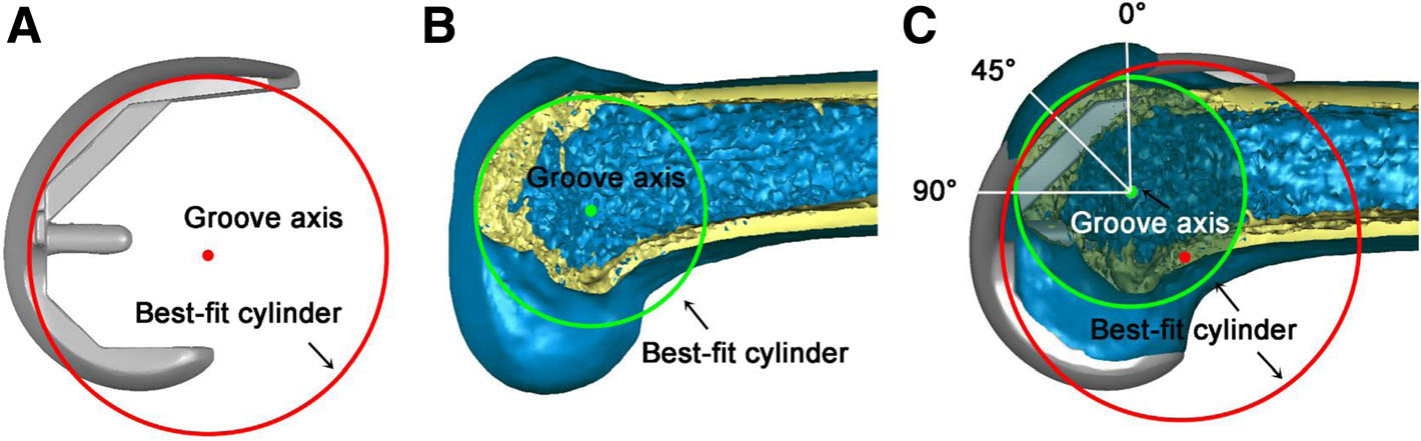
Definition of the three cross-sections of the femoral trochlea in a three-dimensional model of the knee. **a**, **b** To help define the geometrical parameters of interest, a cylinder was established in the sagittal plane, with its axis represented by the axis of the trochlear groove, and its radius adjusted to allow the cylindrical surface to closely fit the trochlear groove of the prosthesis (**a**) or that of the bone (**b**). The fit was first performed by eye, provided that the trochlear groove was clearly visible in both the model of the prosthesis (**a**) and in that of the native knee (**b**). **c** Cutting planes were defined at 0, 45 and 90° starting form a plane parallel to the transverse plane, and rotating in 45° increments towards the distal end of the trochlear groove. The cutting planes were applied to the cylinder whose surface best fit the trochlear groove (**a**, **b**). The axis of the best-fit cylinder for the native knee (*green circle*) corresponds to the groove axis (**b**). The axis of the best-fit cylinder for the prosthetic knee (*red circle*) may not coincide with that of the best-fit cylinder for the native knee (*green*), as the radius was adjusted to allow the cylindrical surface to closely fit the trochlear groove of the prosthesis, leading to variations in sulcus height for the three cross-sections analyzed

**Fig. 3:**
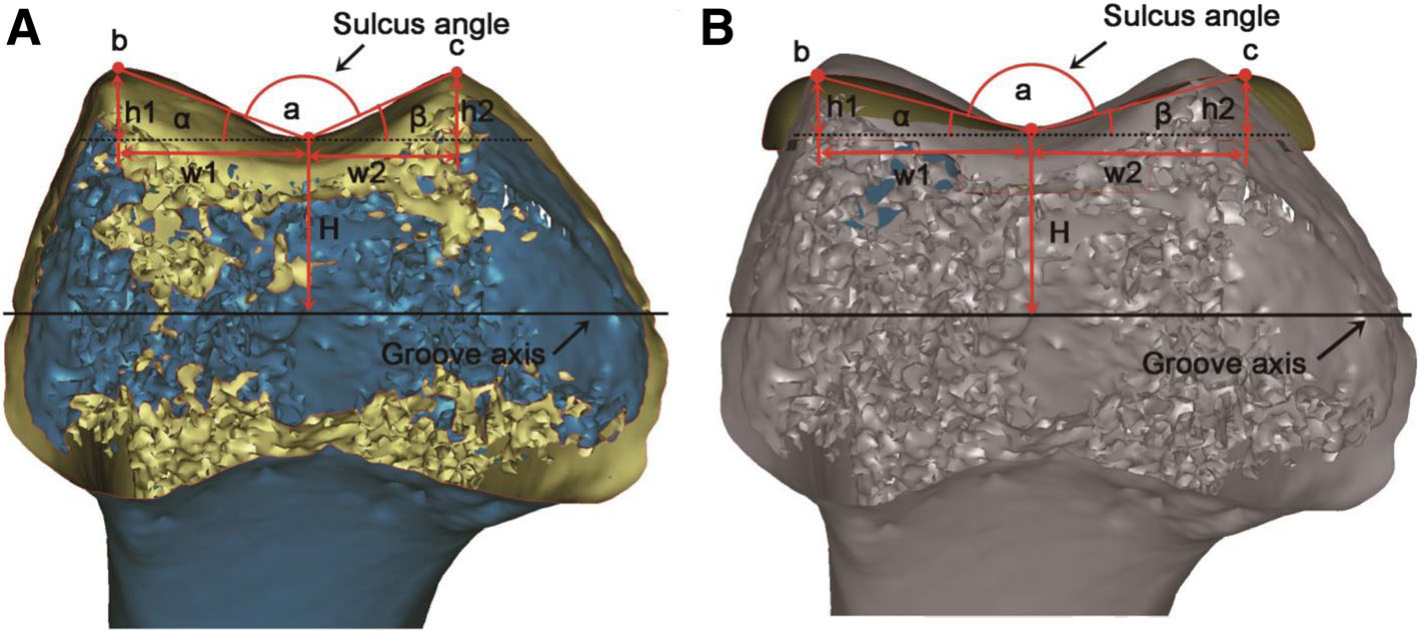
Definition of parameters characterizing the cross-sectional morphology of the femoral trochlea (transverse plane) in three-dimensional models of the native (**a**) and prosthetic knee (**b**). The lateral tilt angle (α) could be calculated as arctan (h1/w1), while the medial tilt angle (β) could be calculated as arctan (h2/w2)

In the sagittal plane, a cylinder was established with its axis parallel to both the coronal and transverse planes, and its radius was adjusted to allow the cylindrical surface to closely fit the trochlear groove of the bone (Fig. 2); the axis of the cylinder was represented by the axis of the trochlear groove (Figs. 2 and 3). The fit of this cylindrical region of interest was first performed by the eye, based on experience, provided that we could clearly identify the groove of the prosthesis and the groove of the native knee (Fig. 2). Then, the radius of the cylinder and the position (coordinates) of its center were recorded; this measurement was performed twice to test the repeatability and sensitivity of the results. Once the cylinder was fitted, the geometrical parameters could be established. Starting from a plane parallel to the transverse plane (0° cutting plane), cutting planes were established in 45° increments towards the distal end of the trochlear groove, resulting in three cross-sections (0, 45 and 90°; Fig. 2). In each cross-section (Fig. 3), the deepest point in the trochlear groove (*a*) and the highest point on each condyle facet (*b*, *c*) were identified in both the native- and prosthetic-knee models. The following parameters characterizing the cross-sectional morphology of the femoral trochlea were defined: sulcus height (H), as the distance between point *a* and the groove axis; lateral tilt angle (α), as the angle between segment *ab* and the groove axis; medial tilt angle (β), as the angle between segment *ac* and the groove axis; sulcus angle, as the angle between segments *ab* and *ac* (Fig. 3). In order to obtain the values of the cross-sectional morphology parameters for angles, the following geometric parameters were defined: *h1* and *h2*, representing the distances between a line parallel to the groove axis that passes through point *a*, and points *b* and *c*, respectively; *w1* and *w2*, representing the distances between sulcus height (H) and *h1* and *h2*, respectively (Fig. 3). Subsequently, the lateral and medial tilt angles were calculated as α = arctan (*h1*/*w1*) and β = arctan (*h2*/*w2*). The morphological parameters were measured or calculated, as appropriate, for each of the 3D models used (i.e., 42 models of the native knee, and 42 × 4 models of prosthetic components).

To eliminate inter-observer bias, all measurements were performed by the same surgeon. To assess the repeatability of the measurements, each parameter was measured two times in one randomly selected model of the native knee. A test-retest analysis was performed to determine intra-observer reliability between the initial measurements and a repeat measurement performed over a month later. The low standard deviation of the two measurements indicated high repeatability of the measurements.

### Statistical analysis

One-way analysis of variance (Fisher’s Least Significant Difference and Student-Newman-Keuls) was used to compare the measurements for different cross-sections. The Student’s t test was used to compare the native and prosthetic knees in terms of sulcus height (H), lateral tilt angle (α), medial tilt angle (β) and sulcus angle in each cross-section. A *p*-value less than 0.05 was considered to indicate statistical significance.

## Results

The APk for all right knees included in the study was 55.4–58.4 mm. Based on the criterion |APk – Apc| < 2 mm, we selected the following components: MP of size 3 (APc = 56.5 mm), Triathlon of size 7+ (APc = 57 mm), NRG of size 7+ (APc = 55.8 mm), and NexGen of size E (APc = 56.9 mm).

**Fig. 4:**
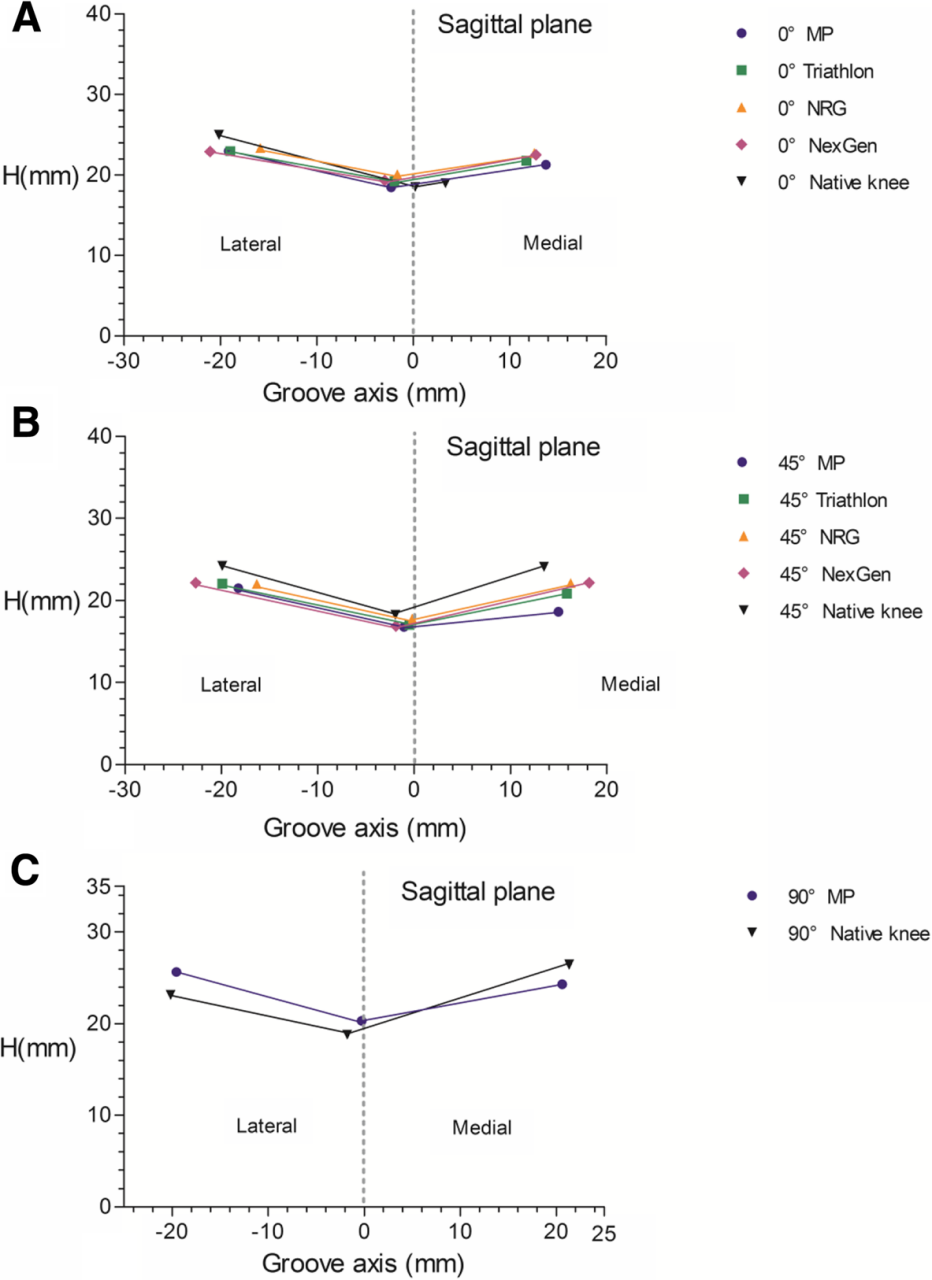
Sulcus height (H) as a key geometric feature of the patellofemoral articular surface. In each case, the line describes the overall profile of the patellofemoral articular surface, characterized by the deepest point in trochlear groove (*a*; *midpoint of the line*) and the highest point on each condyle facet (*b*, *c*; *outer points of the line*). See Fig. 4a for an explanation of each parameter. **a** 0° cross-section; **b** 45° cross-section; **c** 90° cross-section

There was no significant difference among the values measured for sulcus height (H) (average, 18 mm) in the in three cross-sections of the native-knee model (Table 1, Fig. 4). In the 0° cross-section, sulcus height (H) was 18.52 mm in the native knee, which was significantly smaller than that noted for the Triathlon, NRG and NexGen components (*p* < 0.05; Table 1, Fig. 4). Concerning angles, only 28 knee models were included in the analysis, because the beginning of trochlear groove varied significantly with each individual; as such, the trochlear groove, lateral tilt angle (α) and medial tilt angle (β) could not be identified in this cross-section for all models. Compared to the native knees, prosthetic components showed significantly smaller lateral tilt angle (α), and significantly larger medial tilt angle (β) (*p* < 0.05; Table 2, Fig. 4). In the 45° cross-section, the sulcus height (H) was 18.24 mm in the native knee, which was significantly larger than that noted for the MP, Triathlon and NexGen components (*p* < 0.05). Both lateral tilt angle (α) and medial tilt angle (β) were significantly lower in the prosthetic than in the native knees (*p* < 0.05; Table 3, Fig. 4). In the 90° cross-section, only data regarding the MP component were collected, as most prosthetic trochlear grooves of the other knee components (Triathlon, NRG and NexGen) were not long enough to be intersected with the 90° cutting plane (cross section), and point *a* could not be identified in this cross-section for prosthetic components other than MP. For the native knee, sulcus height (H) was 18.78 mm, which was significantly smaller than that noted for the MP component (*p* < 0.05); the native knee showed significantly smaller lateral tilt angle (α) and significantly larger medial tilt angle (β) (*p* < 0.05; Table 4, Fig. 4). In all cross-sections, the sulcus angle was significantly smaller in the native than the prosthetic knees (*p* < 0.05; Tables 2, 3 and 4, Fig. 4).

**Table 1:** Sulcus height (mm) in cross-sections of the femoral trochlea, as measured in threedimensional models of native and prosthetic knees (*n* = 42)

	0° cross-section	45° cross-section	90° cross-section
MP	18.5 (1.4)	16.8 (1.5)^*^	20.3 (1.8)^*^
Triathlon	19.2 (1.4)^*^	17.0 (1.5)^*^	-
NRG	20.1 (1.4)^*^	17.8 (1.5)	-
NexGen	19.3 (1.5)^*^	16.9 (1.5)^*^	-
Native knee	18.5 (1.4)^*a^	18.2 (1.3)^*a^	18.8 (1.2)^*a^

All parameters are reported as mean (standard deviation)

^*^Significant difference (*p* < 0.05) when comparing against the native knee

^a^Native knee taken as reference

**Table 2:** The lateral tilt, medial tilt and sulcus angles of the femoral trochlea calculated based on 0° cross-sections of three-dimensional models of native and prosthetic knees (*n* = 28)

0° cross-section			
	Lateral tilt angle (°)	Medial tilt angle (°)	Sulcus angle (°)
MP	15.5	9.8	172.6
Triathlon	13.3	11.4	173.2
NRG	13.9	12.1	173.5
NexGen	12.5	13.2	172.3
Native knee	16.4 (2.9)	4.5 (5.3)	159.1 (6.7)

All parameters are reported as mean or mean (standard deviation)

Significant differences (*p* < 0.05) between the native and prosthetic knees were noted for all angles evaluated

**Table 3:** The lateral tilt, medial tilt and sulcus angles of the femoral trochlea calculated based on 45° cross-sections of three-dimensional models of native and prosthetic knees (*n* = 42)

45° cross-section			
	Lateral tilt angle (°)	Medial tilt angle (°)	Sulcus angle (°)
MP	15.5	6.8	157.7
Triathlon	15.4	14.3	150.3
NRG	15.5	15.6	148.9
NexGen	14.8	15.4	149.8
Native knee	18.5 (2.6)	20.8 (2.8)	140.7 (4.4)

All parameters are reported as mean or mean (standard deviation)

Significant differences (*p* < 0.05) between the native and prosthetic knees were noted for all angles evaluated

**Table 4:** The lateral tilt, medial tilt and sulcus angles of the femoral trochlea calculated based on 90° cross-sections of three-dimensional models of native and prosthetic knees (*n* = 42)

90° cross-section			
	Lateral tilt angle (°)	Medial tilt angle (°)	Sulcus angle (°)
MP	15.2	11.7	153.1
Native knee	13.5 (2.8)	18.4 (3.2)	148.0 (4.8)

All parameters are reported as mean or mean (standard deviation)

Significant differences (*p* < 0.05) between the native and prosthetic knees were noted for all angles evaluated

## Discussion

In the present study, we performed a detailed comparison of the cross-sectional morphology of the femoral trochlea in native and prosthetic knees. We based our choice of cutting planes on the previous report that 20° of patellofemoral flexion occurs for every 30° of knee flexion [13]. Thus, 0, 45 and 90° cross-sections of the trochlear groove are roughly representative of 0, 67.5 and 135° knee flexion [14]. Importantly, as there are differences in femur geometry parameters between Asian and Caucasian populations [15], and our findings were obtained based on measurements in Chinese subjects, it should be kept in mind that our findings are applicable to the Chinese population, and caution should be exerted when extrapolating our conclusions to non-Asian populations.

In our definition, sulcus height (H) represented the distance between the rotating axis and the trochlear groove. Since we found no significant variation among the three cross-sections in terms of sulcus height (H) values for the native knee models, we concluded that the surface of the cylinder defined in our assessment was indeed a close fit to the trochlear groove (Fig. 2). For the prosthetic knees, sulcus height (H) was comparatively higher in the 0° cross-section (by 0.76 mm), lower in the 45° cross-section (by 1.12 mm), then again higher in the 90° cross-section (by 1.53 mm) (Table 1, Fig. 4). Therefore, the native and prosthetic knees differ in terms of the best cylinder radius that would allow the cylindrical surface to closely fit the trochlear groove (Fig. 2). The discrepancies between the native and prosthetic knees in terms of sulcus height (H) values, which are exemplified in Fig. 2, may be related to different positioning of the patella following TKA; as the position of the patella determines the lever arm of the extensor mechanism, such inappropriate design of the prosthetic components is likely to influence quadriceps efficiency as well as joint reaction forces and contact levels on the femoral trochlea or condyles [11]. Such differences might also cause the component to become anteriorly displaced, as evident in the higher sulcus height (H) values noted for the 0 and 90° cross-sections. Richard et al. [16] reported that, with increasing knee flexion, patellar tilt angle in the sagittal plane was substantially greater in prosthetic than in native knees, which might be related to anterior displacement of the implant. Indeed, Mihalko et al. [17] reported that a 2- and 4-mm build-up in the patellofemoral compartment resulted in flexion loss of 1.8 and 4.4°, respectively. Therefore, prosthetic trochlear design should be modified to avoid irritation of the soft tissue during initial and late knee flexion.

In the native knee, we noted that the sulcus angle first decreased and then increased when moving forward through the cross-sections (i.e., 159.11°, 140.66°, 148.04° in the 0°, 45° and 90° cross-sections, respectively), indicating that the bony structure may give more freedom for the patella to engage into the groove, but may hold the patella during knee flexion. This observation should be considered in the context of the patella-femoral reaction force (PRF), which represents the resultant vector of the quadriceps tendon strain force and the patellar tendon strain force, and is oriented inward in the coronal and axial views. The inward vector of PRF, occurring on the slope of the lateral femoral trochlea, neutralizes the lateral vector produced by the Q-angle of the knee [18, 19]. Therefore, the inward vector of PRF increases with the lateral tilt angle (α), stabilizing the patella. In the 0° cross-section, the PRF is relatively small, and thus the decrease in lateral tilt angle (α) has little effect on the PRF vector or the stability of the patella. However, recent studies revealed that quadriceps forces are highest between 70 and 110° [11, 20, 21]. In the 45° cross-section (67.5° of knee flexion), the PRF is relatively high, and thus the decrease in lateral tilt angle (α) might result in a decrease in the PRF vector and subsequent patellar instability. In the 90° cross-section (135° knee flexion), even though the lateral tilt angle (α) in the MP component is higher than that noted in the native knee, the patella transits over the intercondylar notch [11, 22, 23], and it is the retinacula, rather than the bony structure, that might serve as the main factor maintaining patellar stability. As the patella tilts laterally from 0 to 75° of knee flexion [24], the contact area is mainly on the lateral side; however, the medial tilt (β) might affect patellar tilting during knee flexion, together with the sulcus angle, and does not represent the main factor regulating patellar motion in the 0 and 45° cross sections. Additionally, the sulcus angle was larger in the prosthetic components in all cross-sections, which represents an adverse factor for patellar restraint. Therefore, after TKA, patellar stability might be more dependent on the static and dynamic stability of soft tissues rather than on the bony structure.

The present study showed that the prosthetic trochlear design does not correspond to the morphology of the native trochlea. In mid-flexion, the sulcus height (H), sulcus angle and lateral tilt angle (α) were all significantly lower in the prosthetic components, which might cause lever arm shortening, extensor weakness and decrease in the inward vector of PRF. These changes in anatomy might provide explanations for the clinical prevalence of relative quadriceps weakness [12, 25] and potential patellar dislocation after TKA. Hence, the current prosthetic trochlea might not facilitate patellar motion and quadriceps strength in mid-knee flexion.

In a well-aligned and balanced total knee prosthesis, the resurfaced patella will present a complex 3D movement pattern, broadly similar to that noted in the native knee, as discussed above. The behavior of a particular patellar component is dependent on the surface geometry variables of the mating femoral component, as well as the extrinsic stability provided by muscle and soft tissue support. Articular surface geometries of patellar components vary greatly, and each implant design bears particular advantages, with none being ultimately superior [19]. For example, the majority of currently available patellar components are of the all-polyethylene, dome-shaped type, which may compensate for limited degrees of patellar tilt and rotation by maintaining acceptable contact congruency. Regarding femoral components, on the one hand, the MP, Triathlon and NexGen prostheses evaluated provide deepened central femoral grooves in the 45° cross-section, and the MP component has a distal extension of the trochlear groove. For patella resurfacing or non-resurfacing, such designs would be patella-friendly and provide more stability. On the other hand, all four prostheses provide larger sulcus angles than those in the native knee in all three cross-sections examined; for this reason, we speculate that, other than the native patella, the resurfaced patella would maintain stability better than the non-surfaced patella because of improved contact congruency. By contrast, if undergoing arthroplasty, native knees with similar angles would provide more stability if the native patella were retained.

There were some limitations in this study. First, the data regarding three prosthetic systems were not complete for the 90° cross-sections, as the prosthetic groove of the posterior stabilized component was not long enough to be identified in this cross-section. Therefore, more appropriate components should be included in a future study. Second, the geometry of the cartilage surface differed from that of the bone in the trochlea, although the difference was small [14]. Third, this study only focused on the anatomical parameters of the femoral trochlea obtained from 3D-CT images, to provide some explanations with clinical implications. However, the dynamic performance of the implant and its effect on patellar motion and ligament tension should be studied in the future. Finally, only one implant size was included for each prosthetic component, even though most components are available in several sizes. Nevertheless, the implants were sized according to well-established protocols and criteria (e.g., |APk – APc| < 2 mm) and commonly used based on our experience. Moreover, the study participants were selected from an imaging database with data regarding the lower extremities of 100 healthy Chinese individuals (50 males, 50 females). Furthermore, since implants of different sizes are manufactured in the same shape, our findings regarding shape-specific parameters are relevant even if they were obtained based on one implant size.

## Conclusions

Our study revealed that the discrepancy between the trochlear geometries of the native and prosthetic knee may alter knee mechanics. Nevertheless, these observations should be further investigated through cadaveric, computational or fluoroscopic studies. Our findings can be used to optimize biomechanical guidelines for total knee arthroplasty (patellar resurfacing or non-resurfacing) in Chinese individuals so as to decrease the risk of patellar lateral dislocation, maintain stability and optimize extensor kinematics.

## Abbreviations

3D: Three-dimensional
CT: Computed tomography
PRF: Patella-femoral reaction force
TKA: Total knee arthroplasty
